# Direct and indirect effects of elevated CO_2_ are revealed through shifts in phytoplankton, copepod development, and fatty acid accumulation

**DOI:** 10.1371/journal.pone.0213931

**Published:** 2019-03-14

**Authors:** Anna K. McLaskey, Julie E. Keister, Katherina L. Schoo, M. Brady Olson, Brooke A. Love

**Affiliations:** 1 School of Oceanography, University of Washington, Seattle, Washington, United States of America; 2 Shannon Point Marine Center, Western Washington University, Anacortes, Washington, United States of America; Texas A&M University College Station, UNITED STATES

## Abstract

Change in the nutritional quality of phytoplankton is a key mechanism through which ocean acidification can affect the function of marine ecosystems. Copepods play an important role transferring energy from phytoplankton to higher trophic levels, including fatty acids (FA)—essential macronutrients synthesized by primary producers that can limit zooplankton and fisheries production. We investigated the direct effects of *p*CO_2_ on phytoplankton and copepods in the laboratory, as well as the trophic transfer of effects of *p*CO_2_ on food quality. The marine cryptophyte *Rhodomonas salina* was cultured at 400, 800, and 1200 μatm *p*CO_2_ and fed to adult *Acartia hudsonica* acclimated to the same *p*CO_2_ levels. We examined changes in phytoplankton growth rate, cell size, carbon content, and FA content, and copepod FA content, grazing, respiration, egg production, hatching, and naupliar development. This single-factor experiment was repeated at 12°C and at 17°C. At 17°C, the FA content of *R*. *salina* responded non-linearly to elevated *p*CO_2_ with the greatest FA content at intermediate levels, which was mirrored in *A*. *hudsonica*; however, differences in ingestion rate indicate that copepods accumulated FA less efficiently at elevated *p*CO_2_. *A*. *hudsonica* nauplii developed faster at elevated *p*CO_2_ at 12°C in the absence of strong food quality effects, but not at 17°C when food quality varied among treatments. Our results demonstrate that changes to the nutritional quality of phytoplankton are not directly translated to their grazers, and that studies that include trophic links are key to unraveling how ocean acidification will drive changes in marine food webs.

## Introduction

Increasing CO_2_ concentrations in the atmosphere and ocean due to anthropogenic carbon emissions are causing widespread changes in ocean chemistry that reduce seawater pH and the availability of carbonate ions, a process called ocean acidification (OA). Average surface ocean pH has declined from 8.2 to 8.1 since the industrial revolution, and is expected to decline an additional 0.3–0.4 pH units by 2100 [1]. Across a wide range of marine organisms, reduced pH is generally associated with declines in growth, survival, and reproduction with high variability among and within groups [2,3]. Marine species can also be affected by reduced pH through indirect effects, which require the presence of another species to affect the species of interest [4]. Indirect effects such as altered species interactions [5] and changes in habitat-forming organisms [6] will likely drive many of the ecosystem changes caused by OA.

One important species interaction is the transfer of energy and nutrients from one trophic level to the next [5]. The phytoplankton-copepod link is a critical trophic link in pelagic ecosystems because copepods are the most abundant mesozooplankton and are an important food source for fish larvae. High *p*CO_2_ affects the growth rate and elemental composition of marine phytoplankton in species-specific ways [7] and therefore can alter the quantity and quality of prey available for zooplankton through changes in total production, morphology, macronutrient, and micronutrient composition. Elevated *p*CO_2_ generally increases the C:N and C:P ratios of phytoplankton [8,9] and can also affect their fatty acid content [7]. Fatty acids (FA), and in particular polyunsaturated fatty acids (PUFA), are important macronutrients that are predominantly synthesized by primary producers and are necessary for supporting the growth, survival, and reproduction of aquatic organisms [10]. Laboratory studies on individual phytoplankton species have primarily shown negative effects of increased *p*CO_2_ on PUFAs [11–16] or no significant changes in FA [7,17,18]. Mesocosm studies on natural communities have observed a wide range of responses including declines in PUFAs with increased *p*CO_2_ [19], no effect on FA [20] or increased PUFAs [21,22]. Phytoplankton stoichiometry and FA content are also affected by temperature and the interaction between *p*CO_2_ and temperature [12,13,17], making the effect of OA on food quality difficult to predict.

Many OA studies have found that copepods are generally robust to pH levels predicted for the end of the century [23–27], although some species, life stages [28,29], and populations [30,31] are more sensitive. There is also growing evidence of sub-lethal effects such as changes in respiration, ingestion, and reproductive output, that could have important implications for copepod populations [30,32–34]. Changes in the biochemistry of their prey may also influence copepods because their egg production and hatching are dependent on dietary PUFA, particularly eicosapentaenoic acid (EPA; 20:5ω3) and docosahezaenoic acid (DHA; 22:6ω3) [35], which they cannot produce themselves at ecologically significant rates [36,37].

Initial studies that investigated the effects of OA on phytoplankton-copepod linkages concluded that copepod responses corresponded to *p*CO_2_-induced changes in phytoplankton food quality. The copepod *Acartia tonsa* had decreased development rates, growth rates, and egg production when phytoplankton food quality declined with elevated *p*CO_2_ [15,38,39], but *Acartia grani* were not affected by high *p*CO_2_ when there was no change in phytoplankton food quality [18]. However, recent mesocosm studies have shown more complex responses. The FA content of copepods declined with increased *p*CO_2_ in mesocosms that had reduced phytoplankton FA at elevated *p*CO_2_ [20] and when there was no change in the phytoplankton community’s FA [40]. In another mesocosm, PUFA content of the phytoplankton community increased under elevated *p*CO_2_, copepod grazing declined, and copepod FA content was not affected [22]. In a crossed temperature x CO_2_ study, higher temperature was a much stronger driver than CO_2_, causing altered fatty acid composition and declines in copepod abundance, although changes in the phytoplankton were not measured [41]. These studies show that the effect on copepods depends on the phytoplankton responses, but that there are also other complicating factors that modulate copepod responses. Carefully controlled laboratory studies are an important tool to illuminate the mechanisms that underlie these community responses.

There are a variety of ways increased *p*CO_2_ can affect the phytoplankton-copepod link including direct effects on copepod metabolic costs or behavior, and on phytoplankton abundance or cell size, which can influence copepod grazing. The balance between quality and quantity of prey ingested and the metabolic costs of the copepods will ultimately determine the growth and reproductive output of the copepods. In this study, we investigated the effects of *p*CO_2_ on copepod populations mediated by changes in their prey quality using the copepod *Acartia hudsonica* and the cryptophyte *Rhodomonas salina* as a model system. *A*. *hudsonica* is a temperate-boreal coastal calanoid copepod found in the northwest Atlantic, with its congeners found throughout the world’s oceans. The effects of elevated *p*CO_2_ on this species have not been investigated, but other *Acartia* species show varied responses including increased egg production and faster naupliar development [42], decreased egg production [43], and no changes in survival, body size, egg production, hatching, or development rate [26,44]. We acclimated phytoplankton and copepods to different *p*CO_2_ levels and characterized a wide range of responses in each. We hypothesized that OA-mediated changes in phytoplankton FA would drive changes in copepod reproductive output, and that this indirect pathway would be the primary mechanism through which OA would affect the copepods.

## Methods

This study consisted of two separate experiments in which adult *Acartia hudsonica* were maintained at three target *p*CO_2_ levels, 400, 800, 1200 μatm (pH 7.99, 7.75, 7.61), and were fed *Rhodomonas salina* cultured at those same *p*CO_2_ levels. We characterized the physiology and biochemistry of *R*. *salina* and *A*. *hudsonica* and assessed the reproductive output and larval development of *A*. *hudsonica* at each *p*CO_2_ level before and after acclimation to the treatments. In the first experiment (Exp 12C), the temperature was 12°C; in the second (Exp 17C), it was 17°C; experiments were run sequentially, not concurrently.

### Atmospheric carbon control simulator (ACCS)

Experiments were conducted at the Shannon Point Marine Center (SPMC) in Anacortes, Washington. Control of the carbonate chemistry of all cultures and experiments was achieved using an atmospheric carbon control simulator (ACCS) that has been described in detail [45]. In short, the ACCS combines CO_2_-free air with pure CO_2_ using mass flow controllers to achieve the treatment levels; these air-CO_2_ mixtures are then used to bubble reservoirs of 0.2-μm filtered, UV-exposed natural seawater (salinity 28–32) to equilibrate the seawater to target *p*CO_2_ conditions, and distributed to sealed atmospheric simulation chambers where cultures and experimental vessels are maintained. Gas exchange helps maintain target *p*CO_2_ conditions in these chambers. The *p*CO_2_ of inflowing air-CO_2_ mixtures and outflowing headspace gasses are verified with a Li-COR Li-820 CO_2_ sensor. Equilibration reservoirs were held at experimental temperature in an incubator and atmospheric simulation chambers in a temperature-controlled cold room. Only a single cold room was available so conducting a full temperature x CO_2_ factorial experiment was not possible: the two experiments were run sequentially and are treated herein as separate experiments.

The carbonate chemistry of equilibrated seawater and cultures was verified with discrete total inorganic carbon (C_T_) and spectrophotometric pH measurements taken in triplicate. *R*. *salina* cultures and equilibrated seawater used for water changes were sampled daily, *A*. *hudsonica* cultures were sampled every 1–3 days, and larval development containers were sampled when females were removed and at the end of development tests (described below). C_T_ was measured using an Apollo SciTech analyzer (AS-C3); spectrophotometric pH (total scale) was measured using an Agilent 8453A UV-VIS diode array spectrophotometer and the m-cresol blue method [46]. Full carbonate system parameters were calculated using CO2sys [47] using the constants of Mehrbach et al. [48] refit by Dickson and Millero [49] and the total pH scale. Full details on carbonate chemistry methods have been previously described [45].

### Phytoplankton culturing

*Rhodomonas salina* culture was obtained from Biologische Anstalt Helgoland, Alfred Wegener Institute for Polar and Marine Research (M. Boersma). Phytoplankton culturing for this study required a balance between producing enough biomass daily to feed large numbers of copepods and maintaining carbonate chemistry conditions during phytoplankton growth. This tradeoff guided decisions regarding the growth conditions and required preliminary testing of the carbonate system control. Stock cultures of *R*. *salina* were maintained at experimental temperatures in f/2 enriched seawater following Guillard and Ryther [50]. All seawater for phytoplankton culturing was 0.2-μm sterile filtered, and autoclaved prior to being adjusted to target *p*CO_2_ levels in the equilibration reservoirs. New phytoplankton cultures were inoculated every day in a f/10 growth medium (nutrient concentrations adjusted from Guillard and Ryther 1962) to ensure constant food quality for the copepods and harvested after a four-day growth period on a 14:10 hr L:D cycle. As testing had revealed differences in the growth rates between the two experimental temperatures, initial cell densities were adjusted to 10000 cells ml^-1^ for Exp 12C and 2500 cells ml^-1^ for Exp 17C; the same initial densities were used for all *p*CO_2_ treatments. The freshly inoculated cultures were maintained in atmospheric simulation chambers and constantly bubbled with air-CO_2_ mixtures of the target *p*CO_2_ level. Culture densities and cell sizes were determined daily with a Coulter counter (Beckman Coulter Z2) on five replicate samples, and carbon, nitrogen, and FA content of *R*. *salina* were measured every few days (described below). Specific growth rate *μ* (d^-1^) was calculated from the daily cell counts according to the equation: *μ* = (ln(*D*_*1*_)-ln(*D*_*0*_)/*T*, where *D*_*0*_ is the starting cell density, *D*_*1*_ the final density, and *T* is the growth time (d).

### Copepod culturing and *p*CO_2_ acclimation

*A*. *hudsonica* were obtained from the University of Connecticut (M. Finiguerra, originated from the lab of H.G. Dam). At SPMC, cultures were maintained in water baths at 13–15°C and ambient CO_2_, and fed at surplus from the stock culture of *R*. *salina*. Mature male and female copepods were sorted from the culture over two days prior to the start of experiments. A subset of females were used to test initial egg production, hatching success, and naupliar development (described below); the rest of the adults were distributed into 500-mL jars of *p*CO_2_-equilibrated seawater at a density of 45 individuals per jar, then held in the atmospheric simulation chambers for an acclimation period. During the acclimation period, jars were given a 75% water change daily and fed *p*CO_2_-acclimated *R*. *salina* every 12 hours to maintain a cell concentration above the saturation feeding density of 3000 cells/mL (~0.2 μgC/mL [51]) while allowing for an estimated maximum ingestion rate of 6000 cells/female/hr.

Both experiments had a similar structure that began with a *p*CO_2_ acclimation period starting on day 1; in Exp 12C the acclimation period was six days whereas in Exp 17C, it was reduced to four days due to higher metabolic turnover at higher temperature. *Acartia* sp. are small-bodied, low lipid-storage copepods that have been shown to rapidly respond to food over a period of a few hours [52–54]. In Exp 12C, a subset of females was added to jars of males on day 3 at a ratio of 1 female:2 males to ensure the females would be fertilized for egg production, hatching, and development experiments. In Exp 17C, a subset of females was incubated with males for the entire acclimation period at a ratio of 1 female:2 males. At the end of each acclimation period, females that had been incubating with males were used for egg production and subsequent hatching and naupliar development tests. In Exp 12C, two replicate egg production, hatching, and naupliar development trials were run before and after the acclimation period; however, due to logistical constraints, the 800 μatm *p*CO_2_ target treatment was only included in one trial. Respiration and ingestion rate tests took place over two days (days 6 and 7 in Exp 12C and days 4 and 5 in Exp 17C) and consisted of two replicate respiration tests and two separate ingestion rate tests using acclimated and non-acclimated prey (described below). Remaining females were put in food-free water for 24 hrs before being frozen for FA analyses. Females were not reused in any tests except those that were used in ingestion rate tests were also included in elemental and FA analyses.

### Reproductive output and naupliar development

In all egg production, hatching, and naupliar development tests, females were incubated individually inside mesh-bottom egg production chambers suspended within 250-ml containers of treatment *p*CO_2_-equilibrated seawater held inside the atmospheric simulation chambers. After 24 h, the females were removed, measured for prosome length (head and thoracic segments), and the containers of undisturbed eggs were placed back into the atmospheric simulation chambers to develop. The duration of the tests differed between Exp 12C and Exp 17C because of faster development rates at the higher temperature: in Exp 12C, eggs and nauplii were allowed to develop for 8 days after spawning and in Exp 17C they developed for 5 days.

After hatching, nauplii were fed *R*. *salina* once per day at 25% of the density given adults (described above). During tests of the direct effects of *p*CO_2_ on *A*. *hudsonica* naupliar development, the nauplii were fed stock *R*. *salina* that had not been *p*CO_2_ acclimated; during tests at the end of the acclimation period, nauplii were fed *R*. *salina* that had been cultured at the corresponding target *p*CO_2_ treatment. Nauplii were fed starting on day 4 in Exp 12C and day 2 in Exp 17C so that food would be available when they reached the Nauplius II stage, the first feeding stage. At the conclusion of the naupliar development tests, 10% of the jars were checked for naupliar survival and all were preserved in 5% buffered formalin/seawater solution for counting and staging. Hatching success was calculated from the number of hatched nauplii and unhatched eggs found in each container at the end of the experiment; naupliar development was calculated by the proportion of hatched nauplii that reached the Nauplius IV stage (N IV) at the end of the experiment. The number of females included in each trial varied from 20 to 60 females per treatment.

### Respiration rate

The effects of *p*CO_2_ concentration on the respiration rate of adult female *A*. *hudsonica* was measured in two replicate trials for each experiment with oxygen microsensors. 2-ml vials filled with *p*CO_2_-equilibrated seawater from the corresponding *p*CO_2_ treatment and containing 7 females each were monitored with PreSens Oxygen Sensor Spots (Fibox 4 with PST3 sensor spots, PreSens Precision sensing, Germany) under dim light every 15 minutes for 4.4–5.4 hrs. Each respiration test consisted of five replicate vials containing females and five equilibrated seawater blanks per treatment. Copepods were transferred from the acclimation jars into filtered sterilized seawater before they were added to the respiration vials to reduce the transfer of microbes with them; the same volume of this seawater that was added with the copepods was also added to each blank. After each test, female prosome lengths were measured and respiration rate was standardized to dry weight, calculated from prosome length following the equation of Durbin et al. [55]. During Exp 12C, temperature during respiration rate measurements was 13.5 °C; during Exp 17C, measurements were made at 16.9 °C.

### Ingestion rate

Ingestion rate of *A*. *hudsonica* can be affected directly through *p*CO_2_ effects on the copepods as well as indirectly as a response to *p*CO_2_ effects on the phytoplankton. To separate these two processes, the effect of *p*CO_2_ acclimation on female *A*. *hudsonica* ingestion rate was tested on copepods grazing *R*. *salina* that had been cultured under ambient (400 μatm) *p*CO_2_ regardless of the copepod *p*CO_2_ acclimation treatment, and again on *R*. *salina* cultured at the same acclimation *p*CO_2_ level as the copepods. These two tests were each conducted once per experiment using 250-ml bottles containing 15 females per bottle, with four replicates and two control bottles without copepods per treatment. Initial *R*. *salina* concentrations were ~300 μg C L^-1^ (calculated assuming 75 pg C/cell). Bottles were covered in foil and incubated for 24 hours, after which female copepods were measured; cell concentrations were measured before and after the incubation with a Coulter counter (Beckman Coulter Z2) after being preserved in 5% acid Lugol’s solution. Cell counts were corrected for growth in the control bottles and ingestion rates were calculated according to Frost [56] and standardized to measured prosome length.

### Elemental and fatty acid composition

*R*. *salina* elemental (C, N) and fatty acid composition were evaluated at several time points throughout the experiments; in Exp 12C, 14 elemental samples and 8–9 fatty acid samples were taken per treatment and in Exp 17C, 7–10 elemental and 3–4 fatty acid samples were taken per treatment. *A*. *hudsonica* elemental composition was only measured in Exp 17C; fatty acid composition was evaluated on 3–5 samples per treatment at the end of each experiment. For stoichiometric analysis of carbon and nitrogen content, approximately 4 x 10^6^ phytoplankton cells were filtered onto a pre-combusted GF/F filter and encapsulated in tin foil; *A*. *hudsonica* females that had been starved for 24 hrs were collected in tin capsules (30 copepods per sample). Samples were dried in a drying oven at 60°C for 24 hrs and stored in a desiccator until they were analyzed at the UC Davis Stable Isotope Facility on a PDZ Europa ANCA-GSL elemental analyzer. Phytoplankton samples for FA analysis were filtered as for elemental analysis and stored in Eppendorf tubes layered with N gas and frozen at -80°C until further analysis; copepods for FA analysis were counted into glass test tubes, rinsed with DI water, layered with N gas and frozen at -80°C. Lipids were extracted from the samples using a modification of the methods described by Folch et al. [57] and Bligh and Dyer [58] and the FAs were measured as fatty acid methyl esters (FAMEs); detailed methods are described by Malzahn et al. [59]. Samples for FA analyses were extracted in dichloromethane:methanol (2:1 vol:vol) in an ultrasound bath on ice for 10 minutes and then at -80°C for 24 hours. After centrifugation, the water-soluble fractions were removed by washing with 0.88% KCl buffer. The aqueous phase was discarded and the organic remainder evaporated using N gas. Esterification was achieved by addition of methanolic-sulphuric acid and incubation at 70°C for 75 min. The FAMEs were washed from the methanolic-sulphuric acid using n-hexane. Evaporation of the excess n-hexane yielded the final FAMEs, which were analyzed using a Gas Chromatograph Mass Spectrometer (GC/MS; Varian CP3800 GC with Saturn 2000 Ion Trap MS) equipped with a HP-88 column (0.25mm ID, 30m length, 0.2μm film; Agilent Technologies) at Western Washington University. FAs were identified using a NIST 08 MS library and quantified using a known amount of C19:0 added to each sample at the first extraction step.

### Fatty acid accumulation

We calculated the ratio of *A*. *hudsonica* total FA content to total ingested FA and compared across *p*CO_2_ treatments. Total ingested FA was calculated by multiplying the average *R*. *salina* total FA concentration by the average *A*. *hudsonica* ingestion rate for each treatment and a FA accumulation efficiency was calculated by dividing the average *A*. *hudsonica* total FA concentration by the total ingested FA.

### Statistical analyses

Phytoplankton cell size, carbon and nitrogen content, and C:N ratio were analyzed using linear mixed effects models using sampling date as a random factor with post-hoc least-squares means comparisons among treatments using the R packages lme4 and emmeans. Phytoplankton growth rate, copepod carbon and nitrogen carbon content, phytoplankton and copepod fatty acid content, and copepod respiration and ingestion rate were tested for differences among *p*CO_2_ levels within each experiment using an ANOVA and post-hoc Tukey HSD tests. *A*. *hudsonica* egg production was tested with negative binomial models (glmber.nb) to account for the overdispersion of the data due to females that did not spawn eggs. Female prosome length was considered as a covariate in the models with treatment as a fixed factor and experimental trial as a random factor. The proportion of eggs that hatched and the proportion of hatched nauplii that developed to the N IV stage were tested using mixed effects logistic regressions on a logit scale with the lme4 R package. Experimental trial and individual female brood were included as random effects.

## Results

### Chemistry

Chemistry conditions were generally constant over time within each experiment as well as between the two experiments, and *p*CO_2_ treatments were distinct from each other in both experiments (Table 1). In general, measured pH was slightly higher and calculated *p*CO_2_ was lower than target levels in algal cultures whereas pH was lower and *p*CO_2_ was higher than target levels in copepod-acclimation and naupliar-development jars.

**Table 1:** Water chemistry summary. Average conditions in each experiment grouped by target *p*CO_2_ treatment, ± 1 standard deviation of (n) measurements. Measurements were taken from pre-equilibrated water, algal cultures, adult copepod acclimation jars, and naupliar development rate jars. Salinity, total inorganic carbon (C_T_), and pH were measured; *p*CO_2_ and total alkalinity (A_T_) were calculated.

Exp 12C	Target *p*CO_2_	Salinity	C_T_ (μmol/kg)	pH (total)	*p*CO_2_ (μatm)	A_T_ (μmol/kg)
Equilibrated water	400	28.7 ± 1.9 (9)	1873 ± 9 (9)	7.99 ± 0.01 (9)	420 ± 8 (9)	2005 ± 8 (9)
	800	28.4 ± 1.5 (9)	1954 ± 14 (9)	7.75 ± 0.02 (9)	776 ± 33 (9)	2012 ± 13 (9)
	1200	29.0 ± 1.5 (13)	1979 ± 19 (13)	7.61 ± 0.02 (13)	1100 ± 52 (13)	2000 ± 17 (13)
Algal cultures	400	30.8 ± 1.2 (23)	1992 ± 18 (23)	8.12 ± 0.05 (23)	327 ± 38 (23)	2194 ± 28 (23)
	800	30.9 ± 1 (23)	2095 ± 16 (23)	7.82 ± 0.03 (23)	693 ± 38 (23)	2186 ± 22 (23)
	1200	31.0 ± 1.1 (23)	2142 ± 24 (23)	7.68 ± 0.04 (23)	994 ± 88 (23)	2190 ± 29 (23)
Acclimation jars	400	30.9 ± 0.8 (8)	1946 ± 18 (8)	7.92 ± 0.03 (8)	516 ± 39 (8)	2064 ± 18 (8)
	800	30.8 ± 0.8 (11)	2016 ± 11 (11)	7.71 ± 0.03 (11)	877 ± 59 (11)	2070 ± 14 (11)
	1200	30.8 ± 0.8 (9)	2038 ± 12 (9)	7.58 ± 0.01 (9)	1193 ± 20 (9)	2057 ± 15 (9)
Development jars	400	30.6 ± 0.5 (9)	1938 ± 14 (9)	7.93 ± 0.02 (9)	501 ± 18 (9)	2069 ± 34 (9)
	800	30.7 ± 0.5 (9)	2020 ± 48 (9)	7.71 ± 0.02 (9)	884 ± 69 (9)	2081 ± 56 (9)
	1200	30.8 ± 0.7 (9)	2044 ± 58 (9)	7.58 ± 0.02 (9)	1215 ± 119 (9)	2069 ± 64 (9)
**Exp 17C**						
Equilibrated water	400	31.3 ± 0.9 (12)	1919 ± 13 (12)	7.99 ± 0.01 (12)	435 ± 11 (12)	2096 ± 18 (12)
	800	31.3 ± 0.8 (12)	2008 ± 7 (12)	7.75 ± 0.02 (12)	818 ± 39 (12)	2096 ± 8 (12)
	1200	31.3 ± 0.9 (12)	2051 ± 8 (12)	7.60 ± 0.01 (12)	1185 ± 42 (12)	2094 ± 10 (12)
Algal cultures	400	31.9 ± 0.5 (18)	1979 ± 17 (21)	8.11 ± 0.02 (18)	318 ± 40 (21)	2229 ± 13 (18)
	800	31.7 ± 0.5 (18)	2081 ± 29 (21)	7.88 ± 0.06 (18)	623 ± 91 (21)	2224 ± 15 (18)
	1200	31.9 ± 0.5 (18)	2158 ± 15 (21)	7.70 ± 0.03 (18)	972 ± 74 (21)	2237 ± 9 (18)
Acclimation jars	400	31.1 ± 0.7 (12)	1956 ± 10 (15)	7.96 ± 0.01 (12)	458 ± 45 (15)	2123 ± 10 (12)
	800	31.5 ± 0.5 (12)	2034 ± 25 (15)	7.75 ± 0.03 (12)	819 ± 58 (15)	2129 ± 33 (12)
	1200	31.5 ± 0.5 (12)	2077 ± 20 (15)	7.60 ± 0.02 (12)	1137 ± 108 (15)	2131 ± 13 (12)
Development jars	400	31.0 ± 0 (9)	1969 ± 34 (15)	7.98 ± 0.03 (9)	447 ± 70 (15)	2138 ± 33 (9)
	800	31.3 ± 0.5 (9)	2039 ± 32 (15)	7.81 ± 0.05 (9)	748 ± 74 (15)	2142 ± 43 (9)
	1200	31.3 ± 0.5 (9)	2080 ± 33 (15)	7.65 ± 0.06 (9)	1029 ± 120 (15)	2136 ± 35 (9)

### Phytoplankton

Elevated *p*CO_2_ affected *R*. *salina* physiology broadly through generally increased growth rate, cell size, and carbon content. Growth rate in Exp 17C was 4 and 8% higher when cultured at 800 and 1200 μatm, respectively (p<0.0001; Tukey 400–800 p = 0.013, 400–1200 p<0.0001, 800–1200 p = 0.008), but differences in growth rate in Exp 12C were not statistically significant (p = 0.25). *R*. *salina* cell size was 15 and 14% larger at 800 and 1200 μatm, respectively, than at 400 μatm in Exp 12C and 18 and 16% larger in Exp 17C (Table 2, S2 Table). Carbon content of *R*. *salina* was variable over time and sampling date was a significant random factor in the best model for both experiments (S1 Table). Carbon content was 8 and 5% higher when cultured at 800 and 1200 μatm, respectively, in Exp 12C, and 9 and 8% higher than at 400 μatm in Exp 17C (Table 2, S2 Table). C:N molar ratios also increased by 4 and 2% at 800 and 1200 μatm compared to 400 μatm in Exp 12C, and by 8 and 5% in Exp 17C (Table 2), with sampling date again a significant random factor (S1 Table).

**Table 2:** Phytoplankton growth rate, cell volume, carbon content, and C:N ratio. Average values grouped by target *p*CO_2_ treatment, with the standard deviation of (n) measurements. Cell volume was measured via Coulter counter in five replicate samples from each days’ feeding cultures, growth rate was calculated from the average cell count of those five replicates; C:N samples were taken opportunistically over the course of the experiment (sampled on 7 days in Exp 12C; 4 days Exp 17C).

	Target pCO_2_	Growth Rate (d^-1^)	Cell Vol (μm^3^)	Carbon Content (pg/ cell)	C:N
Exp 12C	400	0.60 ± 0.05 (11)	235 ± 10 (35)^a^	84 ± 13 (14)^a^	5.85 ± 0.46 (14)^a^
	800	0.62 ± 0.04 (10)	271 ± 13 (35)^b^	91 ± 14 (14)^b^	6.10 ± 0.37 (14)^b^
	1200	0.64 ± 0.05 (11)	268 ± 12 (35)^b^	88 ± 19 (14)^ab^	5.99 ± 0.50 (14)^b^
Exp 17C	400	0.96 ± 0.02 (9)^a^	245 ± 14 (40)^a^	75 ± 5 (9)^a^	6.04 ± 0.27 (9)^a^
	800	1.00 ± 0.03 (9)^b^	288 ± 11 (40)^b^	82 ± 4 (7)^b^	6.52 ± 0.39 (7)^b^
	1200	1.04 ± 0.03 (9)^c^	285 ± 16 (40)^b^	81 ± 7 (10)^b^	6.37 ± 0.64 (10)^ab^

Superscript letters indicate statistically significant differences among *p*CO_2_ treatments according to ANOVA and post hoc Tukey test (growth rate) or least-squares means comparisons (cell volume, carbon content, and C:N ratio).

**Fig 1:**
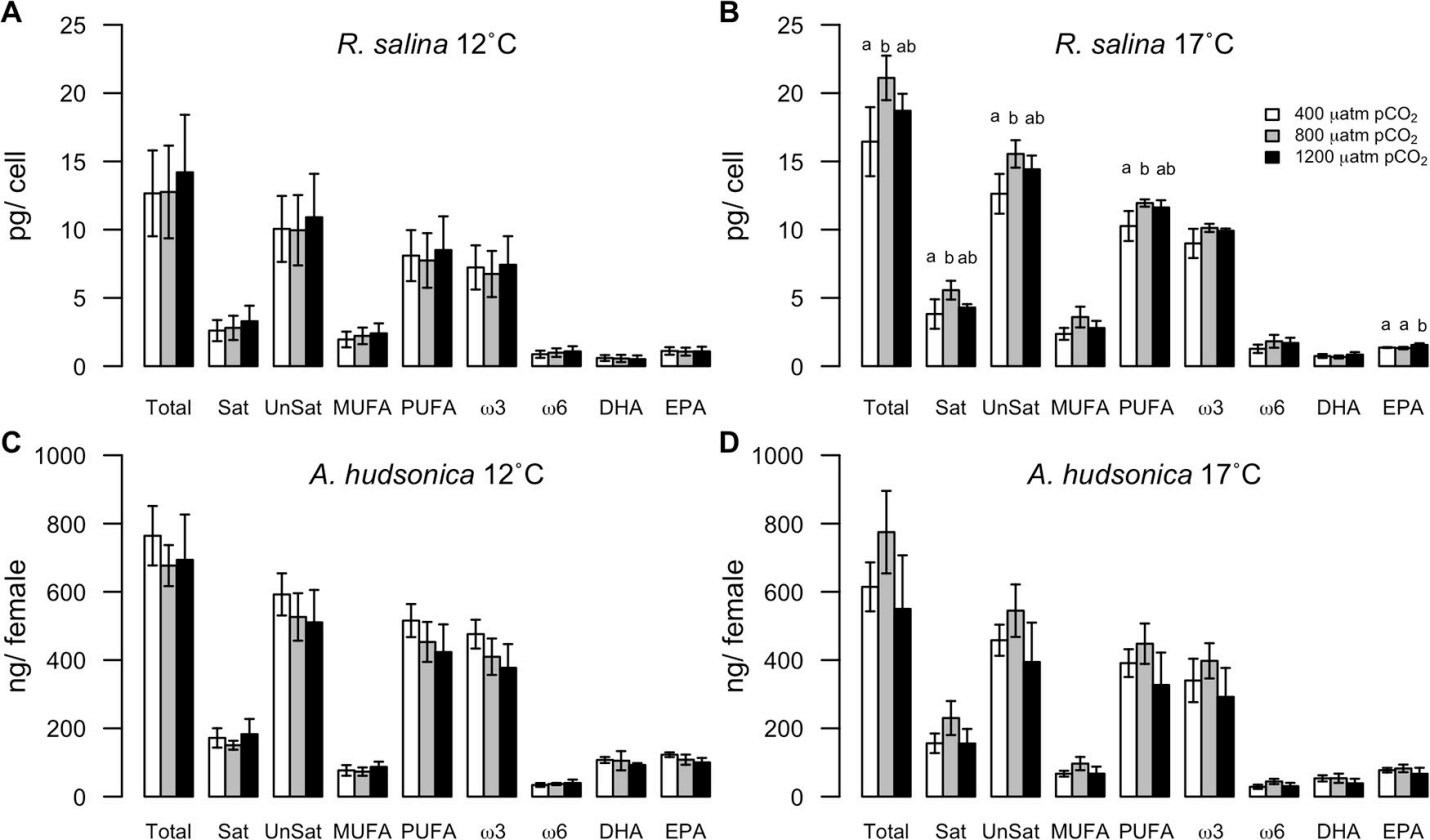
Fatty acid content of *R*. *salina* (A, B; pg/cell) and *A*. *hudsonica* (C, D; ng/female) during Exp 12C and Exp 17C. Error bars show ± 1 standard deviation; letters indicate where significant differences among *p*CO_2_ treatments were detected by Tukey post hoc tests.

*R*. *salina* FAs responded to increased *p*CO_2_ differently between the two experiments with few observed effects in Exp 12C and strong non-linear effects in Exp 17C (Fig 1, S1 Fig). In Exp 12C, there were significant shifts in the proportions of several FA classes but no significant differences in the per cell FA content of *R*. *salina* among *p*CO_2_ treatments (S3 and S4 Tables). At elevated *p*CO_2_ the ratio of ω6:ω3 increased, the proportion of MUFA relative to total FA increased, and the proportion of PUFA decreased. In Exp 17C, a wide range of FAs were greatest in cultures acclimated to 800 μatm *p*CO_2_ (Fig 1, S3 Table). These differences were significant for total FA, saturated, unsaturated, PUFA, and EPA, but there were no differences in the proportions of each FA type (S3 and S4 Tables).

### Copepods

#### Biochemistry

There were no significant differences in carbon content (p = 0.90), nitrogen content (p = 0.89), or C:N (p = 0.28) of *A*. *hudsonica* females raised at different *p*CO_2_ levels in Exp 17C (Table 3). These data are not available from Exp 12C. *A*. *hudsonica* in Exp 12C did not show large differences in FA content with *p*CO_2_ (Fig 1, S3 Table), but the ratio of ω6:ω3 increased with elevated *p*CO_2_, and the proportion of PUFA declined with elevated *p*CO_2_ (S1 Fig, S4 Table). Likewise, in Exp 17C, there were no significant differences in *A*. *hudsonica* FA content with *p*CO_2_ (Fig 1, S3 Table), but copepods acclimated to 800 μatm *p*CO_2_ had significantly higher proportions of MUFA and lower proportions of PUFA (S1 Fig, S4 Table).

**Table 3:** Carbon content, nitrogen content, and C:N of female *A*. *hudsonica* in Exp 17C. Mean and standard deviation of (n) samples containing 30 females each. C and N were not measured in Exp 12C.

Target *p*CO_2_	Carbon Content (μg C/copepod)	Nitrogen Content (μg N/copepod)	C:N
400	3.29 ± 0.22 (5)	0.78 ± 0.03 (5)	4.92 ± 0.17 (5)
800	3.41 ± 0.93 (4)	0.82 ± 0.2 (4)	4.83 ± 0.15 (4)
1200	3.21 ± 0.21 (3)	0.80 ± 0.06 (3)	4.71 ± 0.19 (3)

#### Ingestion rate

**Fig 2:**
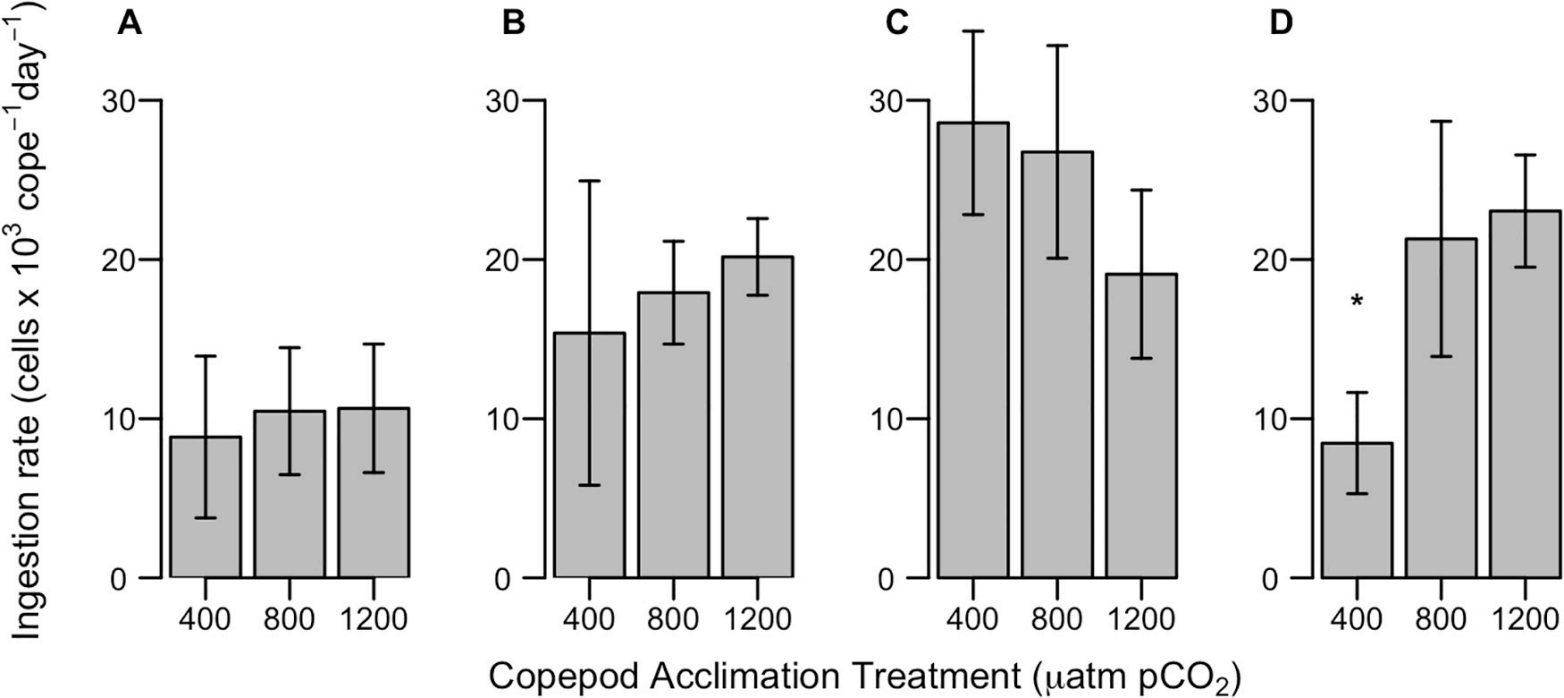
Ingestion rate of *A*. *hudsonica* females on *R*. *salina* acclimated to 400 μatm *p*CO_2_ (A, C) and on *R*. *salina* acclimated to the same target *p*CO_2_ as the copepods (B, D) in Exp 12C (A, B) and Exp 17C (C, D). Error bars show ± 1 standard deviation of four replicates; asterisk indicates where a treatment was significantly different from the other two *p*CO_2_ treatments.

There were no differences among copepod *p*CO_2_ acclimation treatments when the copepods grazed *R*. *salina* that had been cultured under ambient (400 μatm) *p*CO_2_ regardless of the copepod *p*CO_2_ acclimation treatment (Fig 2a and 2c; Exp 12C p = 0.811; Exp 17C p = 0.105). In Exp 12C, copepod ingestion rate was also not affected when females grazed *R*. *salina* acclimated to the same *p*CO_2_ level as the copepods (Fig 2b; p = 0.539). In Exp 17C, *A*. *hudsonica* females had significantly higher ingestion rates in the 800 and 1200 μatm *p*CO_2_ treatments compared to the 400 μatm treatment when grazing *R*. *salina* cultured under those same *p*CO_2_ treatments (Fig 2d; p = 0.005).

#### Respiration rate

**Fig 3:**
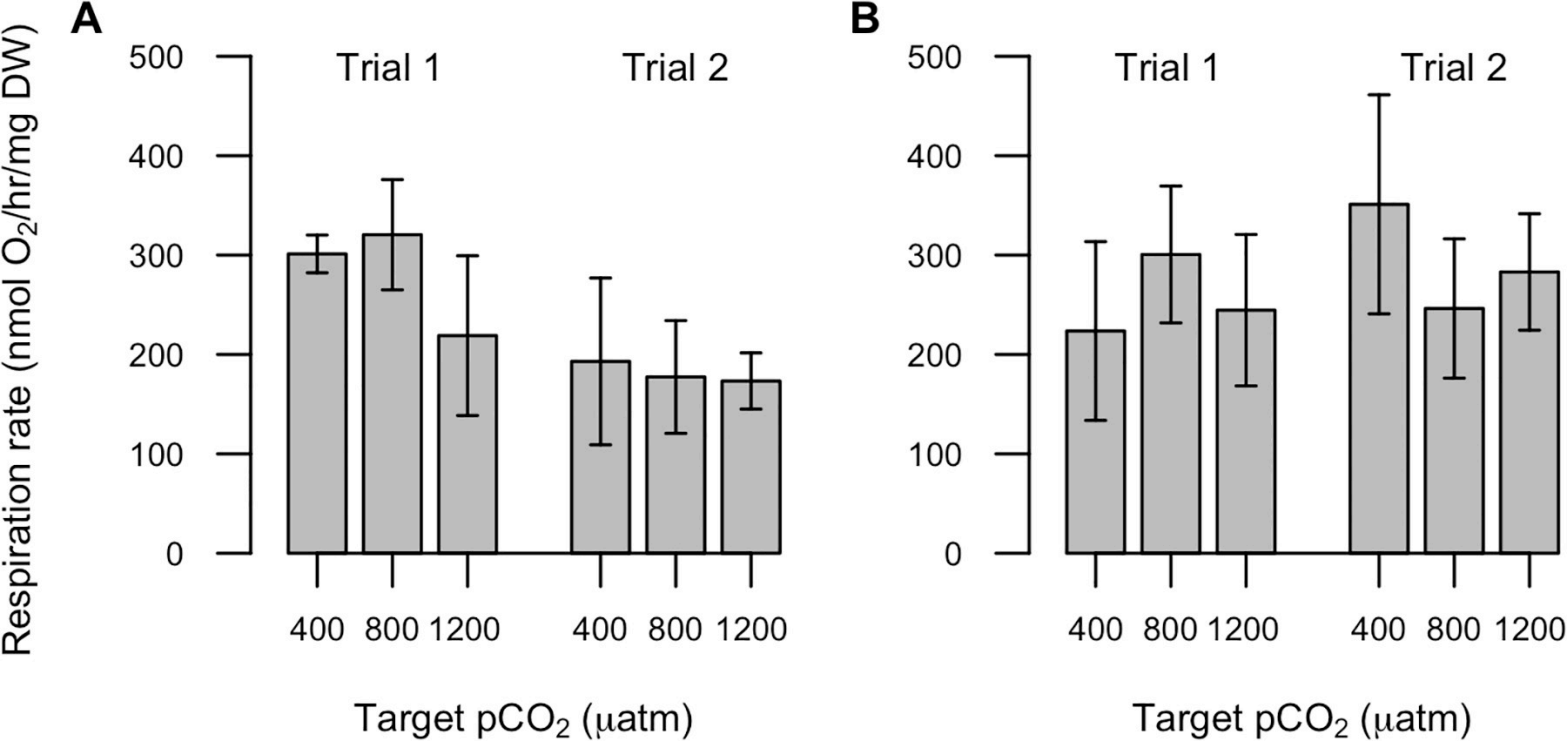
Respiration rate of adult female *A*. *hudsonica* after the *p*CO_2_ acclimation period. The average of five replicates per treatment are plotted for two trials during Exp 12C (A) and Exp 17C (B); error bars show ± 1 standard deviation.

There were no clear effects of *p*CO_2_ on female *A*. *hudsonica* respiration rate in either experiment (Fig 3). Respiration rate was similar among treatments in both trials of Exp 12C (Trial 1 p = 0.06, Trial 2 p = 0.79) and Exp 17C (Trial 1 p = 0.70, Trial 2 p = 0.24).

#### Reproductive output and naupliar development

Egg production and hatching success were both highly variable among females within treatments and among experimental trials. We did not detect effects of *p*CO_2_ on hatching before or after the acclimation periods in either experiment (Table 4, S5 Table). Egg production was only significantly different in Exp 12C pre-acclimation tests, when fewer eggs were produced in the 800 μatm *p*CO_2_ target treatment (Table 4, S6 Table). Due to logistical constraints, the 800 μatm *p*CO_2_ target treatment was only included in one of the trials (both pre- and post-acclimation) of Exp 12C, which had lower egg production and hatching success across all treatments. This was accounted for in the statistical models; however, the values at 800 μatm given in Table 4 are not directly comparable to those in the 400 and 1200 μatm *p*CO_2_ target treatments, which include measurements from both trials.

**Table 4:** Average egg production (eggs/mm female prosome length), proportion hatched, and proportion Nauplius IV (N IV) of individual females’ broods in tests pre- and post-acclimation in Exp 12C and Exp 17C. Average responses of broods in each experiment grouped by target *p*CO_2_ treatment, with the standard deviation of (n) measurements (number of females, broods, and broods with hatching for egg production, hatching, and development, respectively).

	Target pCO_2_	Egg Production (eggs/ mm PL)	Prop Hatch	Prop N IV Stage
12°C Pre	400	3.4 ± 4.2 (52)^a^	0.46 ± 0.41 (32)	0 ± 0 (18)
	800	0.4 ± 0.8 (32)^b^	0.21 ± 0.39 (7)	0 ± 0 (2)
	1200	2.3 ± 3.8 (52)^a^	0.42 ± 0.42 (26)	0.01 ± 0.02 (15)
12°C Post	400	13.8 ± 8.0 (49)	0.50 ± 0.47 (54)	0.20 ± 0.29 (34)^a^
	800	10.7 ± 5.7 (22)	0.18 ± 0.34 (24)	0.21 ± 0.38 (8)^ab^
	1200	14.2 ± 7.4 (56)	0.59 ± 0.46 (60)	0.38 ± 0.31 (40)^b^
17°C Pre	400	10.6 ± 7.9 (48)	0.79 ± 0.30 (45)	0.02 ± 0.05 (42)
	800	7.6 ± 6.0 (44)	0.87 ± 0.19 (44)	0.01 ± 0.04 (43)
	1200	9.8 ± 8.8 (47)	0.80 ± 0.28 (39)	0 ± 0.01 (37)
17°C Post	400	18.3 ± 12.3 (85)	0.22 ± 0.31 (85)	0.30 ± 0.33 (46)
	800	15.6 ± 11.1 (68)	0.20 ± 0.30 (72)	0.27 ± 0.35 (38)
	1200	17.2 ± 12.9 (83)	0.20 ± 0.31 (80)	0.33 ± 0.38 (36)

Superscript letters indicate where significant differences among *p*CO_2_ treatments were detected by Tukey post hoc tests. Note: in Exp 12C, the 800 μatm *p*CO_2_ target treatment was only included in one of two trials so those values are not directly comparable to those in the 400 and 1200 μatm *p*CO_2_ target treatments, which include measurements from both trials.

**Fig 4:**
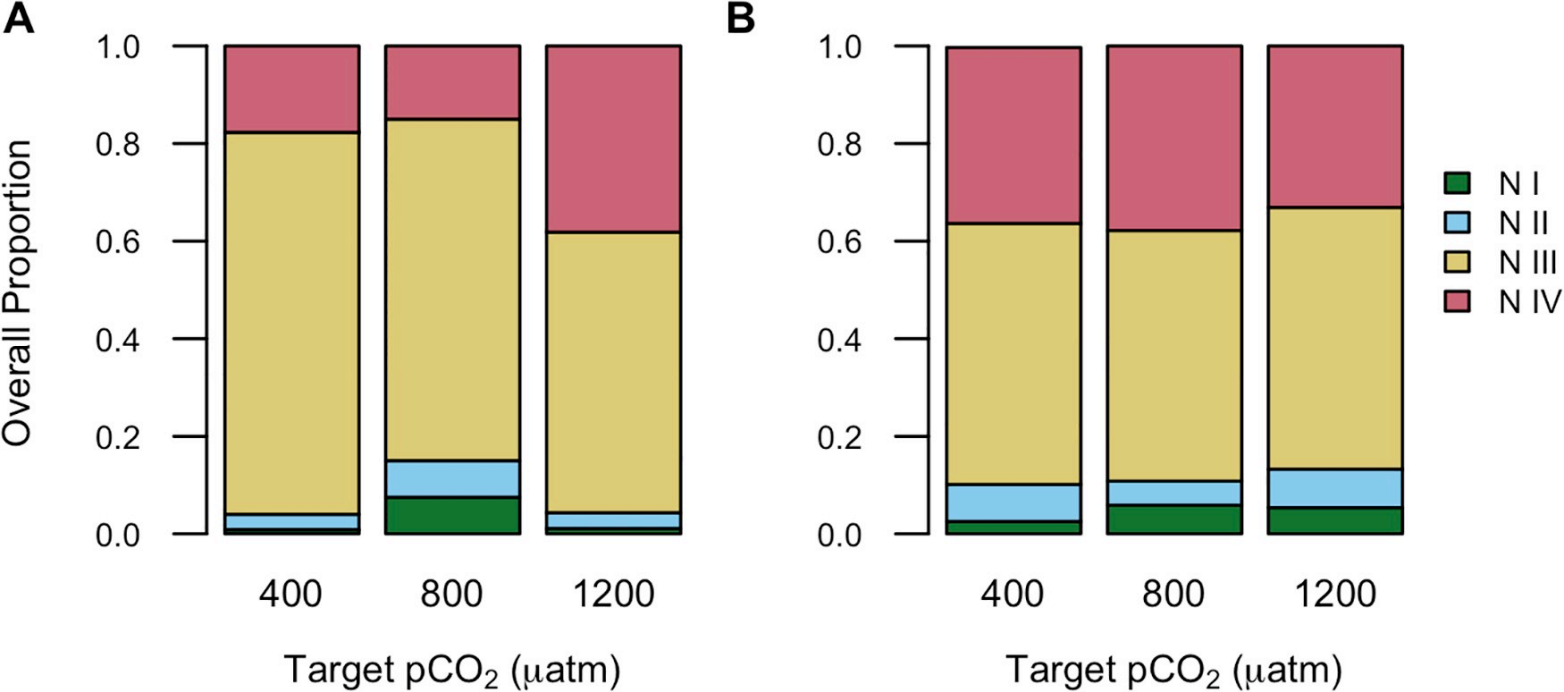
Final proportions (all broods combined) of naupliar stages. Nauplius I (N I), Nauplius II (N II), Nauplius III (N III), and Nauplius IV (N IV) following development tests of eggs spawned from *p*CO_2_-acclimated *A*. *hudsonica* females in Exp 12C (A) and Exp 17C (B).

Higher proportions of nauplii reached the N IV stage in the 1200 μatm *p*CO_2_ treatment compared to 400 μatm in Exp 12C (Fig 4A, S5 Table). In Exp 17C, there were no significant differences in final naupliar proportions among *p*CO_2_ acclimation treatments (Fig 4B, S5 Table). There were no differences in naupliar development with *p*CO_2_ level in pre-acclimation tests (testing the short-term direct effects of *p*CO_2_ only) in either experiment. Naupliar mortality was low (0–2%) in all development tests except for one trial after the acclimation period in Exp 12C that had 15% mortality.

#### Fatty acid accumulation

Differences in total FA content of *R*. *salina* among *p*CO_2_ treatments, combined with different ingestion rates, led to even great differences in the total FA ingested by copepods among *p*CO_2_ treatments. In both experiments, more FA was ingested at elevated *p*CO_2_ and FA accumulation efficiency decreased with increasing *p*CO_2_ level (Table 5).

**Table 5:** Total fatty acids (FA) ingested by *A*. *hudsonica* and FA accumulation efficiency ratios. Total FA ingested was calculated from *R*. *salina* total FA concentrations and *A*. *hudsonica* ingestion rates. FAA is the ratio of *A*. *hudsonica* total FA to total FA ingested.

	Target *p*CO_2_ (μatm)	Total FA ingested (pg/day)	FA Accumulation Efficiency
Exp 12C	400	195,276	3.91
	800	229,265	2.95
	1200	286,245	2.42
Exp 17C	400	138,758	4.42
	800	449,237	1.73
	1200	430,889	1.28

## Discussion

This study set out to determine how copepods are affected by *p*CO_2_-driven changes in phytoplankton food quality. We hypothesized that changes to the trophic pathway would be the primary mechanism by which OA affected copepod reproductive output, but contrary to our expectations, we found that copepod responses were the result of both direct *p*CO_2_ effects and indirect food quality effects. The responses of phytoplankton FA to elevated *p*CO_2_ differed between the two experiments, while the FA patterns of *A*. *hudsonica* generally followed those of their prey. However, *p*CO_2_ also affected naupliar development independent of food quality and elevated *p*CO_2_ caused a shift in how copepods accumulated FA. Our results indicate that both direct and indirect effects of elevated *p*CO_2_ will ultimately determine the outcome for copepod populations.

Phytoplankton are affected by *p*CO_2_ in species-specific ways that can alter the quantity and quality of food available for grazers [7–9]. We attribute differences in the effect of *p*CO_2_ on *R*. *salina* fatty acids between experiments to the different temperatures. Although methodological constraints precluded a single temperature by *p*CO_2_ factorial experiment here, other studies that have directly addressed temperature and *p*CO_2_ as multiple stressors have shown both individual and interactive effects on phytoplankton stoichiometry and FA content [12,13,17,40]. Phytoplankton fatty acids are also affected by other growth conditions such as nutrient supply ratios [17], phytoplankton growth phase [22], and growth rate [60]; although in general, taxonomic differences among groups far outweigh growth conditions in determining phytoplankton FA profiles [61]. Unraveling the responses of phytoplankton to *p*CO_2_ has been a research focus because their differential responses are assumed to be the primary drivers of *p*CO_2_ impacts on the phytoplankton-copepod linkage.

Phytoplankton food quality is an important driver of copepod population dynamics, but is difficult to define and can be characterized in many ways (e.g., macronutrient, lipid, protein, carbohydrate, fatty acid, amino acid, or cholesterol content), with different compounds likely limiting grazer production at different times and under different growth conditions [62]. Elemental stoichiometry is often used as a first approximation of food quality, but can change independently of FA depending on multiple environmental drivers [17], and should not be considered in isolation when evaluating possible effects on grazers. FAs are consistently reported as important indications of energy transfer to higher trophic levels and better determinants of egg production than C:N in the laboratory [63] and field [64,65]. Many studies interpret *p*CO_2_ effects on phytoplankton as changes in food quality without testing the effect on consumers, so characterizing the relative importance of different measures of food quality is essential for understanding the overall effect of *p*CO_2_ on grazers.

The response of copepods to our *p*CO_2_ acclimation treatments may have been decoupled from responses to changes in phytoplankton food quality due to unmeasured direct effects of *p*CO_2_ on their metabolism and physiology, such as decreased digestion efficiency [66] or increased protein synthesis and ion transport [67]. OA increases maintenance metabolic costs for some copepods [68] but this effect may vary between sexes. Respiration rate of male *Acartia tonsa* increases under elevated *p*CO_2_ but is not affected in females [39]. We did not observe an effect of *p*CO_2_ on copepod respiration rate; however, shifts in energy allocation under elevated *p*CO_2_ could change how copepods respond to different nutritional components of their diet. This hypothesis is supported by the decline we observed in FA accumulation efficiency with increased *p*CO_2_. A stable metabolic rate in response to changing *p*CO_2_ concentrations does not mean that *p*CO_2_ has no direct impact on an organism, as OA can cause substantial shifts in energy allocation [67]; these shifts can maintain performance in the short term but may have long-term consequences for the population and can modulate the influence of changing food quality.

Copepods at elevated *p*CO_2_ in both experiments accumulated FA less efficiently than those at ambient *p*CO_2_, which has important implications for the transfer of FA to higher trophic levels. Although this decline in FA accumulation efficiency indicates metabolic shifts in *A*. *hudsonica*, we would have expected to see the same response in both ingestion rate tests in Exp17C if direct effects on the copepods were driving the responses. It is important to note that the low ingestion rates at 400 μatm were only observed in the first ingestion rate trial, despite them feeding on the same food source (400 μatm *R*. *salina*) in each. Copepod ingestion rate can be influenced by many prey characteristics including cell size [56] and food quality [69]. We observed increased cell size at elevated *p*CO_2_ in both experiments but increased FA only at 17°C, so it is possible that different FA content drove the observed differences in ingestion although the ability of copepods to detect and respond to nutritional changes in their prey is not universal or well understood [70].

The most unexpected result in this study was that nauplii developed faster, as indicated by higher proportions of late stage nauplii, at elevated *p*CO_2_ in the 12°C experiment relative to ambient *p*CO_2_, despite a small decline in food quality. Development rate is a complex measure that integrates many physiological processes that could decouple naupliar development rate from food quality. One potential mechanism is maternal provisioning. Exposure of female copepods to elevated *p*CO_2_ conditions can improve the performance of their offspring reared in those conditions [71] and non-linear effects of *p*CO_2_ on reproductive output suggest that under pH stress copepods may reallocate energy from somatic growth towards reproduction [34]. The first naupliar stage of *A*. *hudsonica* is non-feeding and therefore depends entirely on endogenous energy reserves provided by the mother, a process we were unable to measure due to the low biomass of their eggs. Although unexpected, it is also possible that the faster naupliar development rate at elevated *p*CO_2_ was a direct effect of the *p*CO_2_ treatment. A study of *Acartia bifilosa* found the distribution of naupliar stages was older when cultured at reduced pH after 3 days post-spawning, although there were no differences after 4 days [42]. Increased growth rate at low pH has also been observed in sea star larvae and juveniles [72]. Unfortunately, our tests on the effect of *p*CO_2_ on naupliar development prior to the acclimation period failed to capture a distribution of stages (very few nauplii reached the N IV stage), making it difficult to evaluate whether *p*CO_2_ in isolation caused the change in development rate. We cannot identify the mechanism driving increased development rate, but our results indicate that there are important uncharacterized effects of *p*CO_2_ that modulate the influence of food quality on copepod development.

The non-linear changes in phytoplankton FA content that we observed in the 17°C experiment may help explain variable results among previous studies: if only two *p*CO_2_ treatments are compared, the observed effect of *p*CO_2_ (positive, negative, or none) will depend on which part of the organism’s response curve the chosen treatments lie on. Non-linear responses to *p*CO_2_ are likely common and have been observed, e.g., in calcification rates of diverse taxa [73], phytoplankton DMSP concentration [74], phytoplankton carbon content and growth rate [75]. Approximately half of the published studies investigating the effects of *p*CO_2_ on phytoplankton FA to date have used only two *p*CO_2_ treatments and therefore could not have detected non-linear responses. Because of these considerations, more than two *p*CO_2_ treatments should be used, and other growth conditions should be chosen carefully either for environmental realism or to elucidate the interactions among different factors.

## Conclusions

We found that phytoplankton biochemical responses to increased *p*CO_2_ differed between our two experimental temperatures and that copepod responses were a result of both direct *p*CO_2_ effects and indirect food quality effects. At 12°C there was little change in food quality but naupliar development was faster at high *p*CO_2_, while at 17°C phytoplankton food quality increased at moderate *p*CO_2_ but did not translate to benefits for the copepods, demonstrating that organism responses ultimately arise from a combination of both direct and nutritional effects of *p*CO_2_. This hypothesis is also supported by the decline in the ratio of copepod FA stores to ingested FA with elevated *p*CO_2_. This study shows the importance of testing food quality effects on grazers and cautions against a simple extrapolation of phytoplankton biochemistry to higher trophic levels. Carefully designed experimental systems are needed to properly separate direct effects on grazers from the influence of food quality, which has important implications for design and interpretation of many OA experiments.

## Supporting information

S1 Fig Fatty acid proportions of *R*. *salina* (a, b) and *A*. *hudsonica* (c, d) during Exp 1 (12°C; a, c) and Exp 2 (17°C; b, d). Error bars show ± 1 standard deviation; letters indicate where significant differences among *p*CO_2_ treatments were detected by Tukey post hoc tests.
(PDF)

S1 Table Statistical models and AIC scores for generalized linear mixed effects models of *Rhodomonas salina* cell volume, carbon content, nitrogen content, and C:N ratio. Best model’s AIC score is highlighted in bold.
(PDF)

S2 Table P-values for post-hoc least-squares means comparisons among treatments for *R*. *salina* cell volume, carbon content, and C:N. (PDF)

S3 Table Quantity of fatty acids in *Rhodomonas salina* (pg/cell) and *Acartia hudsonica* (ng/female) during Exp 1 (12 °C) and Exp 2 (17 °C). (PDF)

S4 Table P values for ANOVA and post-hoc Tukey test for fatty acids. Only those with significant ANOVA p values are shown.
(PDF)

S5 Table Statistical models and AIC scores for generalized linear and mixed effects models of *A*. *hudsonica* hatching proportions and proportion of nauplii to develop to the Nauplius IV (N IV) stage. Best model’s AIC score is highlighted in bold.
(PDF)

S6 Table Statistical models and AIC scores for mixed-effects negative binomial models of *A*. *hudsonica* egg production. Best model’s AIC score is highlighted in bold.
(PDF)
