# Supplementary material for: Direct and indirect effects of elevated CO2 are revealed through shifts in phytoplankton, copepod development, and fatty acid accumulation
Source: PLoS One. 2019 Mar 14;14(3):e0213931. doi: 10.1371/journal.pone.0213931 (PMC6417711; doi:10.1371/journal.pone.0213931)
Supplement: S1 Fig — Error bars show ± 1 standard deviation; letters indicate where significant differences among pCO2 treatments were detected by Tukey post hoc tests. (PDF) [file pone.0213931.s001.pdf]

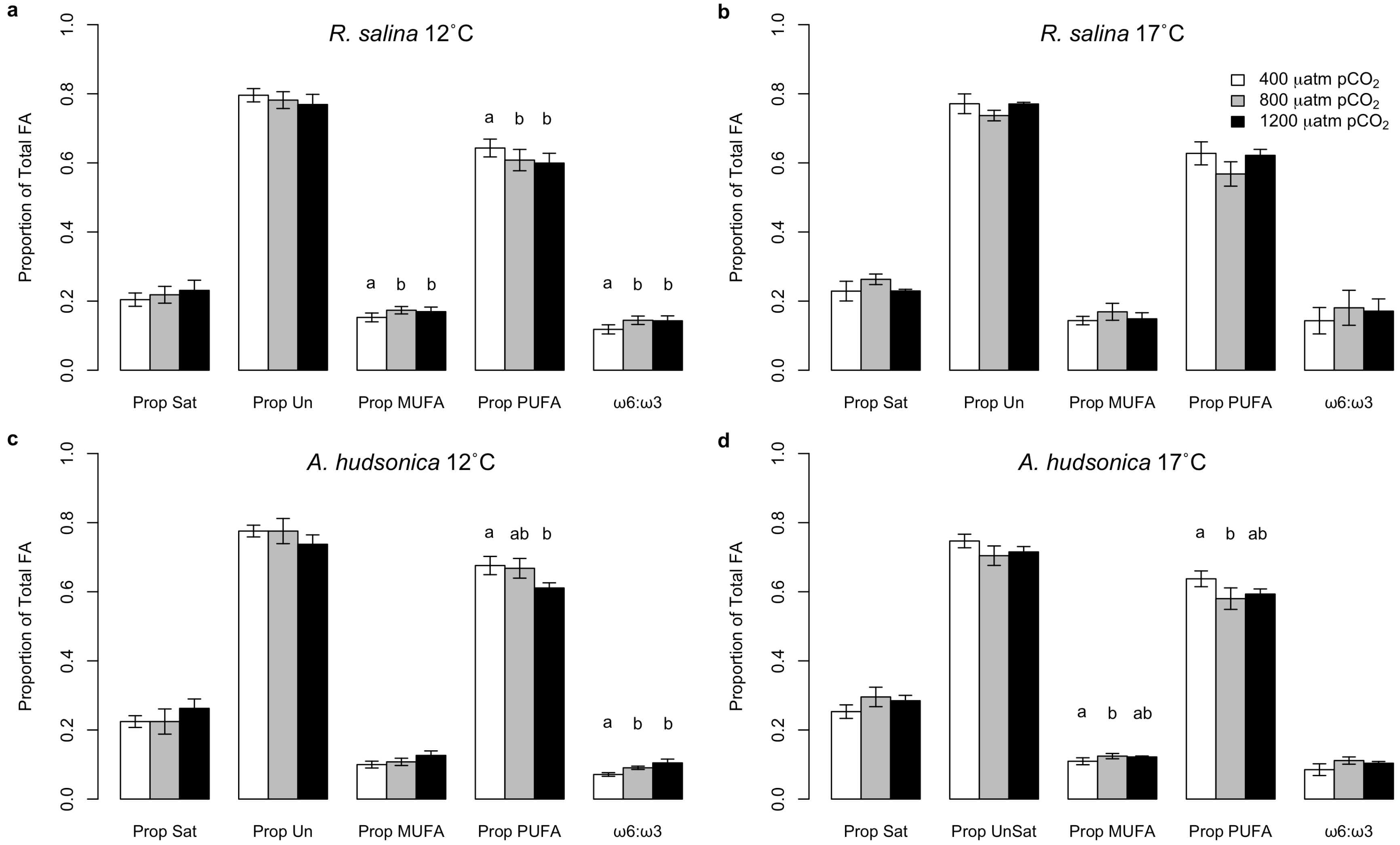

Figure S1. Fatty acid proportions of *R. salina* (a, b) and *A. hudsonica* (c, d) during Exp 12C (a, c) and Exp 17C (b, d). Error bars show ± 1 standard deviation; letters indicate significant differences detected among pCO_2 treatments by Tukey post hoc tests.
