# Supplementary material for: Direct and indirect effects of elevated CO2 are revealed through shifts in phytoplankton, copepod development, and fatty acid accumulation
Source: PLoS One. 2019 Mar 14;14(3):e0213931. doi: 10.1371/journal.pone.0213931 (PMC6417711; doi:10.1371/journal.pone.0213931)
Supplement: S1 Table — Best model’s AIC score is highlighted in bold. (PDF) [file pone.0213931.s002.pdf]

S1 Table. Statistical models and AIC scores for generalized linear mixed effects models of *Rhodomonas salina* cell volume, carbon content, nitrogen content, and C:N ratio. Best model's AIC score is highlighted in bold.

**Experiment 1 (12 C)**	**Cell Volume**	**Carbon Content**	**Nitrogen Content**	**C:N**
Response=Treatment*(Date)	801	304.21	263.61	-4.88
Response=Treatment+(Date)	**801**	**304.21**	263.61	**-4.88**
Response=(Date)	931.55	308	**261.9**	7.63

**Experiment 2 (17 C)**				
Response=Treatment*(Date)	920.57	169.94	139.85	27.99
Response=Treatment+(Date)	**920.57**	**169.94**	139.85	**27.99**
Response=(Date)	1103.98	175.02	**137.73**	36.12
