# Supplementary material for: Direct and indirect effects of elevated CO2 are revealed through shifts in phytoplankton, copepod development, and fatty acid accumulation
Source: PLoS One. 2019 Mar 14;14(3):e0213931. doi: 10.1371/journal.pone.0213931 (PMC6417711; doi:10.1371/journal.pone.0213931)
Supplement: S2 Table — (PDF) [file pone.0213931.s003.pdf]

S2 Table. P-values for post-hoc least-squares means comparisons among treatments for *R. salina* cell volume, carbon content, and C:N.

Contrast	Exp 12C Cell Vol	Exp 12C Carbon	Exp 12C C:N	Exp 17C Cell Vol	Exp 17C Carbon	Exp 17C C:N
400-800	<0.0001	0.021	0.0003	<0.0001	0.024	0.0028
400-1200	<0.0001	0.18	0.038	<0.0001	0.031	0.0638
800-1200	0.47	0.57	0.16	0.31	0.92	0.23
