# Supplementary material for: Direct and indirect effects of elevated CO2 are revealed through shifts in phytoplankton, copepod development, and fatty acid accumulation
Source: PLoS One. 2019 Mar 14;14(3):e0213931. doi: 10.1371/journal.pone.0213931 (PMC6417711; doi:10.1371/journal.pone.0213931)
Supplement: S3 Table — (PDF) [file pone.0213931.s004.pdf]

S3 Table. Quantity of fatty acids in *Rhodomonas salina* (pg/cell) and *Acartia hudsonica* (ng/female) during Exp 12C and Exp 17C.

	12C Rhodomonas (pg/cell)			17C Rhodomonas (pg/cell)			12C Acartia (ng/female)			17C Acartia (ng/female)		
**Fatty Acid**	**400 (n=9)**	**800 (n=9)**	**1200 (n=8)**	**400 (n=4)**	**800 (n=4)**	**1200 (n=3)**	**400 (n=3)**	**800 (n=3)**	**1200 (n=3)**	**400 (n=4)**	**800 (n=4)**	**1200 (n=3)**
C14:0	0.53 ± 0.19	0.55 ± 0.21	0.66 ± 0.27	0.81 ± 0.23	1.27 ± 0.2	0.96 ± 0.08	24.5 ± 7.7	20.3 ± 1.8	21.5 ± 5.6	16.4 ± 3.5	26.5 ± 6	14.5 ± 5
C15:0	0.03 ± 0.02	0.03 ± 0.02	0.04 ± 0.02	0.05 ± 0.01	0.07 ± 0.01	0.06 ± 0.04	1.9 ± 0.5	1 ± 0.9	2 ± 0.9	2.3 ± 0.5	3.9 ± 1.3	2.1 ± 1
C16:0	1.7 ± 0.5	1.9 ± 0.6	2.3 ± 0.8	2.5 ± 0.7	3.6 ± 0.5	2.8 ± 0.1	114 ± 18	100 ± 12	118 ± 24	103 ± 22	154 ± 31	108 ± 27
C16:1n9	0.42 ± 0.14	0.32 ± 0.2	0.45 ± 0.24	0.36 ± 0.3	0.42 ± 0.34	0.48 ± 0.17	0 ± 0	0.9 ± 1.6	2.8 ± 2.7	7 ± 5	12.4 ± 4.6	8 ± 4.6
C16:1n7	0 ± 0	0.1 ± 0.2	0.04 ± 0.12	0.21 ± 0.32	0.56 ± 0.23	0.09 ± 0.15	15.1 ± 6.1	9.4 ± 4.3	11.4 ± 2.9	5.4 ± 4.1	8.6 ± 1.9	6.1 ± 2.6
C17:0	0.16 ± 0.07	0.18 ± 0.05	0.18 ± 0.09	0.25 ± 0.06	0.29 ± 0.02	0.23 ± 0.01	9.5 ± 1.1	8.4 ± 0.2	8.8 ± 1.2	8.6 ± 1.2	10.7 ± 1.7	7.2 ± 3.5
C17:1n7	0.01 ± 0.02	0.01 ± 0.02	0.01 ± 0.02	0 ± 0	0.03 ± 0.03	0 ± 0	7.4 ± 1.7	5.5 ± 4.9	7 ± 0.8	3.9 ± 2.8	6.9 ± 1.3	5.8 ± 0.7
C18:0	0.12 ± 0.06	0.12 ± 0.03	0.13 ± 0.03	0.2 ± 0.05	0.25 ± 0.05	0.23 ± 0.03	22.4 ± 1.6	20.6 ± 1.3	32.5 ± 16.6	26.4 ± 3.1	34.1 ± 11.6	24.1 ± 2.1
C18:1n9 t	0.07 ± 0.05	0.11 ± 0.04	0.12 ± 0.06	0.12 ± 0.02	0.12 ± 0	0.11 ± 0.01	2.1 ± 0.9	2.3 ± 0.9	4.6 ± 2	4.3 ± 0.5	5.8 ± 1.6	3.6 ± 0.5
C18:1n9 c	0.83 ± 0.25	1.09 ± 0.27	1.11 ± 0.35	0.93 ± 0.31	1.59 ± 0.49	1.4 ± 0.59	10.4 ± 1.7	14.6 ± 1.8	16.4 ± 4.2	17.2 ± 7.3	29.7 ± 7.9	17.1 ± 6
C18:1n7	0.62 ± 0.17	0.6 ± 0.17	0.68 ± 0.22	0.75 ± 0.13	0.88 ± 0.05	0.73 ± 0.05	40.2 ± 5.6	37.4 ± 1.7	43.5 ± 17.4	28.8 ± 4.6	33.3 ± 4.2	26.8 ± 8.2
C18:2n6	0.66 ± 0.2	0.75 ± 0.23	0.83 ± 0.3	1 ± 0.2	1.5 ± 0.3	1.5 ± 0.2	22.3 ± 4.1	23.6 ± 0.7	27.1 ± 7.9	19.4 ± 4.6	32.1 ± 4.9	21.7 ± 7.3
C18:3n6	0.14 ± 0.04	0.16 ± 0.05	0.17 ± 0.06	0.13 ± 0.09	0.2 ± 0.13	0.09 ± 0.16	5.6 ± 1	6.2 ± 0.4	6.1 ± 1.3	21.8 ± 34.6	6.9 ± 1.6	4.7 ± 1.2
C18:3n3	2.1 ± 0.4	2.1 ± 0.5	2.4 ± 0.6	2.9 ± 0.4	3.5 ± 0.1	3.3 ± 0.2	97.6 ± 10.7	87 ± 7.2	80.9 ± 20.5	72.3 ± 46.7	110.1 ± 14.3	81.3 ± 23.4
C18:4n3	3.4 ± 0.8	3 ± 0.8	3.5 ± 1	4 ± 0.8	4.6 ± 0.3	4.2 ± 0.2	148 ± 19	110 ± 12	104 ± 32	137 ± 22	151 ± 17	105 ± 32
C20:2	--	--	--	--	--	--	5.6 ± 0.6	6.4 ± 2.3	6.5 ± 2.5	4.8 ± 1	5.8 ± 1.7	4.1 ± 1.4
C20:4n6	0.07 ± 0.03	0.08 ± 0.04	0.08 ± 0.04	0.11 ± 0.02	0.13 ± 0.02	0.14 ± 0.02	6.1 ± 1	7.2 ± 2.2	6.6 ± 1	4.8 ± 0.9	5.4 ± 1.5	4.2 ± 1.2
C20:5n3	1.1 ± 0.3	1.1 ± 0.3	1.1 ± 0.3	1.4 ± 0	1.3 ± 0.1	1.6 ± 0.1	123 ± 7	108 ± 15	100 ± 13	77.4 ± 6.9	82.5 ± 11.3	66.9 ± 20.9
C22:6n3	0.6 ± 0.21	0.56 ± 0.27	0.51 ± 0.28	0.74 ± 0.14	0.67 ± 0.11	0.84 ± 0.19	107 ± 9	105 ± 28	93 ± 5	53.4 ± 9	53.7 ± 13.6	38.8 ± 16.4
C23:0	0.02 ± 0.02	0.01 ± 0.01	0 ± 0	0 ± 0	0.1 ± 0	0 ± 0	--	--	--	0 ± 0	0.6 ± 1.2	0 ± 0
C22:1n9	--	--	--	--	--	--	1.5 ± 1.4	3 ± 2.1	1.3 ± 1.2	0.4 ± 0.8	0 ± 0	0 ± 0
Total	12.7 ± 3.1	12.8 ± 3.4	14.2 ± 4.2	16.4 ± 2.5	21.1 ± 1.6	18.7 ± 1.2	764 ± 87	677 ± 60	694 ± 133	614 ± 72	775 ± 121	550 ± 163
Saturated	2.6 ± 0.8	2.8 ± 0.9	3.3 ± 1.1	3.8 ± 1.1	5.6 ± 0.7	4.3 ± 0.2	172 ± 28	150 ± 13	183 ± 45	156 ± 29	230 ± 50	156 ± 38
Unsaturated	10.1 ± 2.4	10 ± 2.6	10.9 ± 3.2	12.6 ± 1.5	15.5 ± 1	14.4 ± 1	592 ± 62	526 ± 70	511 ± 95	458 ± 46	545 ± 77	394 ± 124
ω3	7.2 ± 1.6	6.8 ± 1.7	7.4 ± 2.1	9 ± 1.1	10.1 ± 0.3	9.9 ± 0.2	476 ± 42	410 ± 53	377 ± 70	340 ± 64	398 ± 52	292 ± 93
ω6	0.87 ± 0.26	0.99 ± 0.31	1.08 ± 0.38	1.3 ± 0.3	1.8 ± 0.5	1.7 ± 0.4	34 ± 5.6	36.9 ± 3.1	39.8 ± 9.9	46 ± 33.9	44.4 ± 7.6	30.6 ± 9.8
MUFA	2 ± 0.6	2.2 ± 0.6	2.4 ± 0.7	2.4 ± 0.4	3.6 ± 0.8	2.8 ± 0.5	76.7 ± 15.5	73.1 ± 12.1	87.1 ± 15.1	67.1 ± 8.5	96.8 ± 19.4	67.4 ± 20.8
PUFA	8.1 ± 1.9	7.7 ± 2	8.5 ± 2.5	10.3 ± 1.1	11.9 ± 0.3	11.6 ± 0.5	516 ± 48	453 ± 59	424 ± 81	391 ± 41	448 ± 59	327 ± 104
DHA/EPA	0.52 ± 0.09	0.5 ± 0.13	0.44 ± 0.14	0.54 ± 0.1	0.51 ± 0.05	0.54 ± 0.1	0.87 ± 0.05	0.96 ± 0.13	0.93 ± 0.09	0.69 ± 0.13	0.65 ± 0.11	0.57 ± 0.08
Sat/Unsat	0.26 ± 0.03	0.28 ± 0.04	0.3 ± 0.05	0.3 ± 0	0.4 ± 0	0.3 ± 0	0.29 ± 0.03	0.29 ± 0.06	0.36 ± 0.05	0.34 ± 0.04	0.42 ± 0.06	0.4 ± 0.03
n6/n3	0.12 ± 0.01	0.14 ± 0.01	0.14 ± 0.01	0.14 ± 0.04	0.18 ± 0.05	0.17 ± 0.04	0.07 ± 0.01	0.09 ± 0	0.1 ± 0.01	0.15 ± 0.15	0.11 ± 0.01	0.1 ± 0
Prop Saturated	0.2 ± 0.02	0.22 ± 0.02	0.23 ± 0.03	0.23 ± 0.03	0.26 ± 0.02	0.23 ± 0	0.22 ± 0.02	0.22 ± 0.04	0.26 ± 0.03	0.25 ± 0.02	0.3 ± 0.03	0.28 ± 0.02
Prop Unsaturated	0.8 ± 0.02	0.78 ± 0.02	0.77 ± 0.03	0.77 ± 0.03	0.74 ± 0.02	0.77 ± 0	0.78 ± 0.02	0.78 ± 0.04	0.74 ± 0.03	0.75 ± 0.02	0.7 ± 0.03	0.72 ± 0.02
Prop MUFA	0.15 ± 0.01	0.17 ± 0.01	0.17 ± 0.01	0.14 ± 0.01	0.17 ± 0.02	0.15 ± 0.02	0.1 ± 0.01	0.11 ± 0.01	0.13 ± 0.01	0.11 ± 0.01	0.12 ± 0.01	0.12 ± 0
Prop PUFA	0.64 ± 0.03	0.61 ± 0.03	0.6 ± 0.03	0.63 ± 0.03	0.57 ± 0.04	0.62 ± 0.02	0.68 ± 0.03	0.67 ± 0.03	0.61 ± 0.01	0.64 ± 0.02	0.58 ± 0.03	0.59 ± 0.01
