# Supplementary material for: Direct and indirect effects of elevated CO2 are revealed through shifts in phytoplankton, copepod development, and fatty acid accumulation
Source: PLoS One. 2019 Mar 14;14(3):e0213931. doi: 10.1371/journal.pone.0213931 (PMC6417711; doi:10.1371/journal.pone.0213931)
Supplement: S4 Table — Only those with significant ANOVA p values are shown. (PDF) [file pone.0213931.s005.pdf]

S4 Table. P values for ANOVA and post-hoc Tukey test for FA. Only those with significant ANOVA p values are shown.

	Rhodomonas Exp 12C				Rhodomonas Exp 17C			
Fatty Acid	ANOVA	400-800	400-1200	800-1200	ANOVA	400-800	400-1200	800-1200
C20:5n3					0.018	0.81	0.044	0.019
Total					0.028	0.023	0.33	0.29
Saturated					0.036	0.034	0.72	0.15
Unsaturated					0.025	0.021	0.18	0.47
PUFA					0.029	0.029	0.098	0.84
ω6/ω3	0.0004	0.0009	0.002	0.97				
Prop MUFA	0.004	0.004	0.026	0.76				
Prop PUFA	0.01	0.04	0.012	0.81				
	Acartia Exp 12C				Acartia Exp 17C			
ω6/ω3	0.0045	0.045	0.0037	0.13				
Prop MUFA					0.042	0.048	0.095	0.9
Prop PUFA	0.032	0.91	0.037	0.062	0.019	0.019	0.066	0.72
