# Supplementary material for: Direct and indirect effects of elevated CO2 are revealed through shifts in phytoplankton, copepod development, and fatty acid accumulation
Source: PLoS One. 2019 Mar 14;14(3):e0213931. doi: 10.1371/journal.pone.0213931 (PMC6417711; doi:10.1371/journal.pone.0213931)
Supplement: S5 Table — Best model’s AIC score is highlighted in bold. (PDF) [file pone.0213931.s006.pdf]

S5 Table. Statistical models and AIC scores for generalized linear and mixed effects models of *A. hudsonica* hatching proportions and proportion of nauplii to develop to the Nauplius IV (N IV) stage. Best model's AIC score is highlighted in bold.

	**12C Pre-acclimation**		**12C Post-acclimation**		**17C Post-acclimation**	
	Prop Hatch	Prop N IV	Prop Hatch	Prop N IV	Prop Hatch	Prop N IV
Response=(Experiment) + (Brood) + Treatment	185.71	22.392	329.58	323.73	892.01	375.61
Response=(Experiment) + Treatment	232.06	20.392	656.36	428.74	2302.53	440.04
Response=(Brood) + Treatment	192.11	20.392	453.82	**321.73**	890.01	373.84
Response=(Brood)	189.85	**16.497**	473.28	325.95	**887.29**	**370.16**
Response=(Experiment)	228.94	**16.497**	656.14	470.76	2300.28	437.78
Response=(Experiment) + (Brood)	**181.71**	18.497	**326.21**	327.95	889.29	371.86

	**17C Pre-acclimation**	
	Prop Hatch	Prop N IV
Response=(Brood) * Treatment	386.2	73.78
Response=(Brood) + Treatment	386.2	73.78
Response=(Brood)	**384.21**	**72.96**
