# Supplementary material for: Direct and indirect effects of elevated CO2 are revealed through shifts in phytoplankton, copepod development, and fatty acid accumulation
Source: PLoS One. 2019 Mar 14;14(3):e0213931. doi: 10.1371/journal.pone.0213931 (PMC6417711; doi:10.1371/journal.pone.0213931)
Supplement: S6 Table — Best model’s AIC score is highlighted in bold. (PDF) [file pone.0213931.s007.pdf]

S6 Table. Statistical models and AIC scores for mixed-effects negative binomial models of *A. hudsonica* egg production. Best model's AIC score is highlighted in bold.

	12C Pre-acclimation	12C Post-acclimation	17C Post-acclimation
Response=(Experiment) + Treatment + ProsomeLength	**439.19**	829.2	1694.49
Response=(Experiment) + Treatment	463.76	913.43	1761.14
Response=(Experiment) + ProsomeLength	451.13	**828.25**	**1691.31**
Response=(Experiment)	472.22	912.66	1758.72

	17C Pre-acclimation
Response=Treatment + ProsomeLength	850.07
Response=Treatment	885.83
Response=ProsomeLength	**850.16**
